# Quantification of mutant E-cadherin using bioimaging analysis of *in situ* fluorescence microscopy. A new approach to *CDH1* missense variants

**DOI:** 10.1038/ejhg.2014.240

**Published:** 2014-11-12

**Authors:** João Miguel Sanches, Joana Figueiredo, Martina Fonseca, Cecília Durães, Soraia Melo, Sofia Esménio, Raquel Seruca

**Affiliations:** 1 Institute for Systems and Robotics and Department of Bioengineering from the Instituto Superior Técnico, Technical University of Lisbon, Lisbon, Portugal; 2 Department of Cancer Genetics, IPATIMUP - Institute of Molecular Pathology and Immunology of the University of Porto, Porto, Portugal; 3 Faculty of Medicine of the University of Porto, Porto, Portugal

## Abstract

Missense mutations result in full-length proteins containing an amino acid substitution that can be neutral or deleterious, interfering with the normal conformation, localization, and function of a protein. A striking example is the presence of *CDH1* (E-cadherin gene) germline missense variants in hereditary diffuse gastric cancer (HDGC), which represent a clinical burden for genetic counseling and surveillance of mutation carriers and their families. *CDH1* missense variants can compromise not only the function of E-cadherin but also its expression pattern. Here, we propose a novel method to characterize E-cadherin signature in order to identify cases with E-cadherin deregulation and functional impairment. The strategy includes a bioimaging pipeline to quantify the expression level and characterize the distribution of the protein from *in situ* immunofluorescence images. The algorithm computes 1D (dimension intensity) radial and internuclear fluorescence profiles to generate expression outlines and 2D virtual cells representing a typical cell within the populations analyzed. Using this new approach, we verify that cells expressing mutant forms of E-cadherin display fluorescence profiles distinct from those of the wild-type cells. Mutant proteins showed a significantly decrease of fluorescence intensity at the membrane and often abnormal expression peaks in the cytoplasm, reflecting the underlying molecular mechanism of trafficking deregulation. Our results suggest employing this methodology as a complementary approach to evaluate the pathogenicity of E-cadherin missense variants. Moreover, it can be applied to a wide range of proteins and, more importantly, to diseases characterized by aberrant protein expression or trafficking deregulation.

## Introduction

Functional E-cadherin is usually processed at the endoplasmic reticulum, continuously transported to the plasma membrane, and recycled through the Golgi apparatus.^1, 2^ In a normal setting, E-cadherin molecules concentrate at the cell membrane, where they establish a homophilic binding to other E-cadherin molecules on neighboring cells.^3, 4^ Simultaneously, the cytoplasmic domain of E-cadherin forms a complex with catenins strengthening cell–cell adhesion and, consequently, supporting the structural and mechanical properties of epithelial tissues.^4, 5, 6^

The presence of *CDH1* mutations, such as in cancer, causes E-cadherin loss of function because of protein absence or aberrant localization.^7, 8^ In hereditary diffuse gastric cancer (HDGC, OMIM: 137215), germline E-cadherin/*CDH1* (OMIM: 192090) mutations are the only causative events known to date.^9, 10, 11^ Pathogenic germline missense variants of E-cadherin often result in decreased E-cadherin expression at the plasma membrane and/or aberrant expression at the cytoplasm.^12, 13, 14^ Therefore, visual inspection of E-cadherin in cell populations by immunofluorescence (IF) is a mandatory approach to depict protein expression. However, *in situ* IF analysis is not a quantitative methodology and is strongly operator-dependent, being the classification based on subjective criteria. Thus, it became imperative applying a quantitative method to examine *in situ* IF images.

Here, we designed an algorithm that computes at one dimension (1D) a representative profile of protein level of expression and distribution in cell populations. To illustrate the biomedical value of the method, we analyzed *in situ* IF images of cells expressing wild-type (WT) E-cadherin or a panel of relevant germline E-cadherin missense variants associated with gastric cancer.^12, 13, 15, 16, 17^ Importantly, this new approach calibrates the data taking into account morphological variability of the cell population because E-cadherin may impact cytoskeleton organization and, in consequence, cell morphology.^4, 18^ We show that the method is able to quantify and map the expression of E-cadherin at the membrane and throughout the cytoplasm, using internuclear (IN) and radial (RD) fluorescence profiles of cells expressing WT and E-cadherin variants, even in the presence of cell heterogeneity.

## Material and methods

### Cell lines

A panel of E-cadherin missense variants that were proven to be functionally relevant has been selected.^12, 15, 16, 18, 19, 20, 21, 22, 23^ As a control, one variant that does not affect E-cadherin function (neutral variant) was also chosen.^17^ Negative-E-cadherin CHO (Chinese Hamster Ovary) cells were transfected with vectors encoding either the WT E-cadherin (reference sequence NM_004360.3) or the variants c.820G>A (p.Gly274Ser), c.1018A>G (p.Thr340Ala), c.1108G>T (p.Asp370Tyr), c.1901C>T (p.Ala634Val), c.2245C>T (p.Arg749Trp), c.2269G>A (p.Glu757Lys), c.2343A>T (p.Glu781Asp), c.2396C>G (p.Pro799Arg), and c.2494G>A (p.Val832Met), as described previously.^14, 17, 24^

### Immunofluorescence

Cells were grown to at least 80% confluence, fixed and stained for E-cadherin. E-cadherin was tagged using a specific antibody (BD Biosciences, Erembodegem, Belgium; mouse, 1:100) and a subsequent fluorescent secondary antibody (Alexa Fluor 488 goat anti-mouse, Invitrogen, Grand Island, NY, USA). Nuclei were counterstained with DAPI. Images were acquired on a Carl Zeiss Apotome Axiovert 200 M Fluorescence Microscope (Carl Zeiss, Jena, Germany) with an Axiocam HRm camera, under a × 40 objective. All experiments were confirmed in three biological replicas.

### Software development and analytical parameters

A software application was developed specifically to assist the operator in the selection of the cells within the plates. The application automatically segments each selected nucleus by combining the Otsu and Watershed methods.^25, 26, 27, 28^ In each IF image, pairs of cells were selected for analysis in a semi-automated manner, allowing the intervention of the user. The process consists of selecting the nucleus of a cell (point one) and subsequently selecting the nucleus of the second cell (point two). The algorithm automatically draws a line joining the two points (one in each nucleus), and crossing the cytoplasm and the plasma membrane of both cells. A large number of pairs of cells can be then connected, and all the data saved.

The mapping and quantification of the protein expression level was performed by computing, respectively, 1D IN and RD intensity profiles of two contiguous cells and within one single cell. To cope with cell size and shape variability, a geometric compensation algorithm was developed in a Bayesian framework. The method was designed as an iterative algorithm composed by the following steps: (i) profile extraction from selected single cells (in case of RD) or pairs of cells (in case of IN); (ii) image map building by stacking fluorescence profiles together in columns after length normalization; (iii) denoising of map image as described by Rodrigues *et al.*^29^ (in which multiplicative noise described by a Poisson distribution is assumed); (iv) geometric compensation of each 1D column profile minimizing the overall variability of the map along the lines (horizontal direction);^30^ and (v) computation of the average and standard deviation profiles using the compensated map. After the extraction of the data, the maximum mean ratio (MMR) parameter was calculated dividing the maximum fluorescence value (numerator) by the fluorescence mean (denominator).

### Statistical analyses

Quantitative parameters of IN profiles (normalized to a constant length of 100 arbitrary units) in WT and mutant cells were analyzed using a Mann-Whitney test with a Bonferroni correction.

## Results

**Figure 1:**
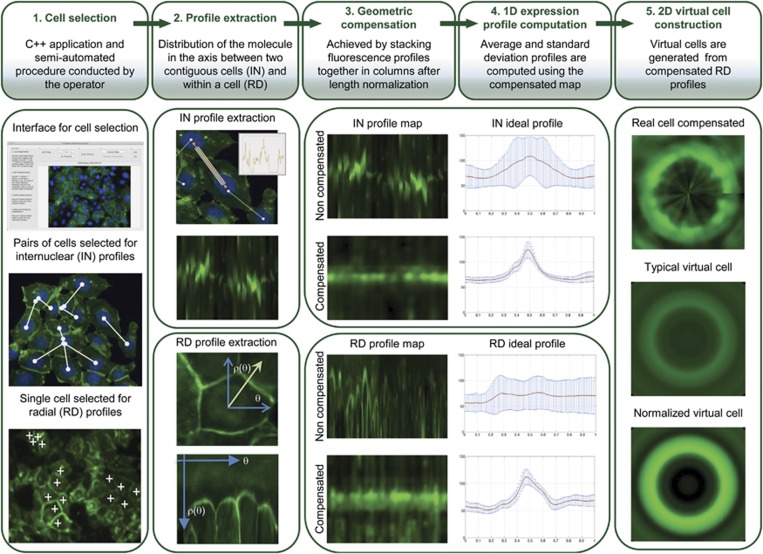
Scheme representing the analytical pipeline. The analytical pipeline includes the following steps: (**1**) cell selection using a C++ application; (**2**) IN and RD profiles extraction; (**3**) geometrical compensation of IN and RD profiles to cope with cell shape and size variability; (**4**) original and compensated IN and RD profiles with corresponding standard deviation bar for each point; and (**5**) 2D virtual cell construction based on RD compensated profiles.

In this work, we propose a novel bioimaging strategy to extract 1D fluorescence intensity profiles (IN and RD) and to construct 2D virtual typical cells from *in situ* IF images. This method grants a rigorous and quantitative description of the level and pattern of expression of a specific protein among cell populations. The complete pipeline describing in detail the different steps of the process is presented in Figure 1.

Original IF images from cell populations expressing WT and different E-cadherin variants were used to extract RD and IN fluorescence profile maps from single cells and pairs of cells. During the process of acquisition of RD and IN profiles of the different cells, two main technical difficulties were found: (i) segmentation of the cell boundaries; in most cases, not clearly observed because of loss or decreased level of E-cadherin expression at the cell membrane, a common event observed in the case of pathogenic variants;^31^ and (ii) cell population variability concerning cell size and shape. To circumvent the first difficulty, the selection of cells within the images followed a semi-automated procedure conducted by the operator in order to only extract the information with true biological meaning. Before map building, the profiles were normalized to a constant length of 100 (arbitrary units) to achieve a method resistant to geometric variability of the cells.

### IN fluorescence intensity profiles characterize E-cadherin expression along contiguous cells

IN profiles were obtained to measure the average expression level of the protein between pairs of neighboring cells. A special focus was given to the plasma membrane where E-cadherin exerts its adhesive function. The IN profiles were able to capture the typical protein distribution along the medial axis of cell pairs, corresponding to the cytoplasm, allowing quantification and mapping of aberrant foci of expression.

We verified that parallel intensity profiles along the axis of several pairs of cells were different because of heterogeneity of cell morphology and differences in the nucleus position. To compensate for these variations, a geometric alignment algorithm was applied. The results demonstrate that a compensated map displays an almost constant horizontal linear pattern of fluorescence, representing E-cadherin expression at the cell membrane. When compared with the non-compensated profile, the compensated one presents a smaller variance at each location and a higher sharpness of the peak, demonstrating the accuracy of the proposed method to map and quantify the level of expression of a specific tag in a cell population.

### RD fluorescence intensity profiles characterize E-cadherin expression in single cells

RD profiles were developed to map, in single cells, the expression level of the protein at the cytoplasm located outside of the IN axis and, therefore, impossible to be captured by the IN profiles. Several RD profiles were extracted analyzing a number of angles, anchored at the geometrical centers of the nuclei of selected cells. As observed in the IN profile, the RD compensated profile shows that a compensated map displays an almost constant pattern of fluorescence when compared with the non-compensated one. Further, the average profile presents a sharp peak that accurately represents the overall distribution of E-cadherin within a cell. A special attention was given to aberrant cytoplasmic E-cadherin expression, as abnormal accumulation of the protein could indicate impairment of its normal localization and function.

Using RD geometric compensated profiles, we were able to reconstruct a 2D virtual cell. This cell represents the level and mapping of E-cadherin expression, and illustrates the typical single cell of a large cell population, excluding intrinsic differences in cell morphology. To improve the visualization of the spatial distribution of the protein, a virtual cell with non-scaled intensity and its contrast enhanced version was generated.

Our results demonstrate that 2D virtual cell images are representative models of a specific protein expression pattern at the plasma membrane and at the cytoplasm in a single cell. These images can be very advantageous to identify patterns of E-cadherin expression distinct from that of the standard cells (WT).

### E-cadherin variants display distinct expression profiles

**Figure 2:**
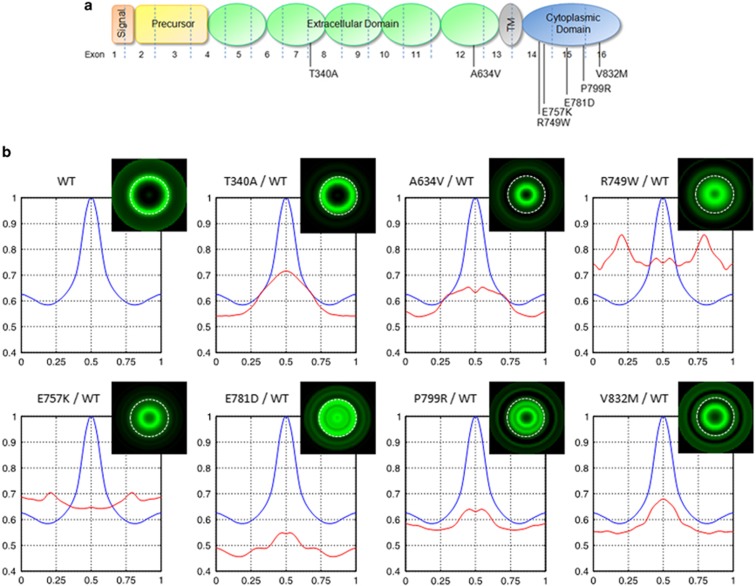
E-cadherin variants display distinct E-cadherin expression profiles. (**a**) Representation of E-cadherin sites affected by the missense variants. The location of the signal peptide, precursor sequence, extracellular domain, transmembrane domain (TM), and cytoplasmic domain are illustrated. (**b**) Average intensity IN profiles of each E-cadherin variant (red line) overlapped with the WT one (blue line). Typical virtual cells for WT and E-cadherin variants are presented.

To test whether the methodology was able to discriminate between the expression pattern of WT and E-cadherin variants, we applied the technique to eight cell lines, one control expressing the normal protein (WT) and seven cell lines expressing different E-cadherin missense variants.^14^ The selected variants span the full length E-cadherin: two are extracellular, two juxtamembrane, and three cytoplasmic variants (Figure 2a). These variants were discovered in the context of HDGC and previously tested for functionality (Table 1 and Supplementary Table 1). All of them have proved to be functionally relevant *in vitro*, impairing the ability of E-cadherin to mediate cell–cell adhesion and to suppress invasion.^12, 13, 15, 16, 18, 19, 20, 21, 22, 23^

IF was performed and a number of images were acquired for each condition. Representative pairs of cells were selected from the images.

We analyzed the fluorescence intensity at the membrane, and the MMR of fluorescence of the different cell lines (Table 2). The MMR quantifies the sharpness of the fluorescence peak at the membrane. High MMR values are associated with a high level and regular pattern of expression at membrane, and with a low level of aberrant protein expression within the cytoplasm.

When compared with the WT IN profile, we verify that all variant cases showed statistically significant decreased fluorescence intensity at the membrane (position 0.5 on the *x* axis, Figure 2b and Table 2). Moreover, a switch of protein localization from the membrane to a concentrated peak at cytoplasm was observed for the variants c.2245C>T (p.Arg749Trp) and c.2269G>A (p.Glu757Lys). In these cases, the highest fluorescence intensity is at positions 0.80 and 0.79, possibly corresponding to the endoplasmic reticulum, localized at the perinuclear region of the cell. This result corroborates our previous findings demonstrating that variants c.2245C>T (p.Arg749Trp) and c.2269G>A (p.Glu757Lys) are retained in the endoplasmic reticulum and induce protein trafficking deregulation (Table 1).^12, 14^ In fact, both variants are remarkable examples of the biological value of our methodology.

The values of MMR in WT E-cadherin cells were significantly higher than those of cells expressing any of the variants (Table 2), a feature associated not only with the high level of protein expression localized at the membrane, but also with the proficient adherens junctions, where E-cadherin is regularly concentrated. Accordingly, MMR results reveal that all E-cadherin variants exhibit a weaker cell–cell adhesion than that established by WT E-cadherin cells.

Analyzing 2D virtual cells that were generated using RD profiles, we verify that the WT cells show an empty circular pattern of fluorescence with a clear concentration of the protein at the peripheral limit, without diffuse expression inside the cell (Figure 2b). This expression pattern illustrates a population of cells in which E-cadherin is mainly located at the plasma membrane, without abnormal protein accumulations in the cytoplasm. A similar result was obtained for the c.1018A>G (p.Thr340Ala) extracellular variant. This variant pattern is in accordance with the results obtained for the IN profile, and likely represents the presence of protein at the membrane without altering its localization. In contrast, all the other mutant proteins show an almost fulfilled circular pattern of fluorescence suggestive of diffuse protein distribution throughout the cell interior, and absence of the protein at the peripheral limit of the virtual cell. This is the representative model of cells displaying loss of E-cadherin at the plasma membrane, and presenting abnormal cytoplasmic accumulations. Within the cytoplasm, the position of protein accumulation may vary depending on the organelle where the protein is retained because of its altered trafficking (eg, endoplasmic reticulum, golgi, endosomes, and lysosomes).

Importantly, 2D virtual cells are not quantitative outcomes, and should be interpreted as qualitative analyses. For quantitative purposes, fluorescence intensity profiles and the MMR must be evaluated.

Overall, the virtual representation of cells based on RD profiles allows a straightforward recognition of variants inducing protein mislocalization, when compared with the WT context. This strategy can thus be indicative of the possible pathogenic significance of new missense variants.

**Figure 3:**
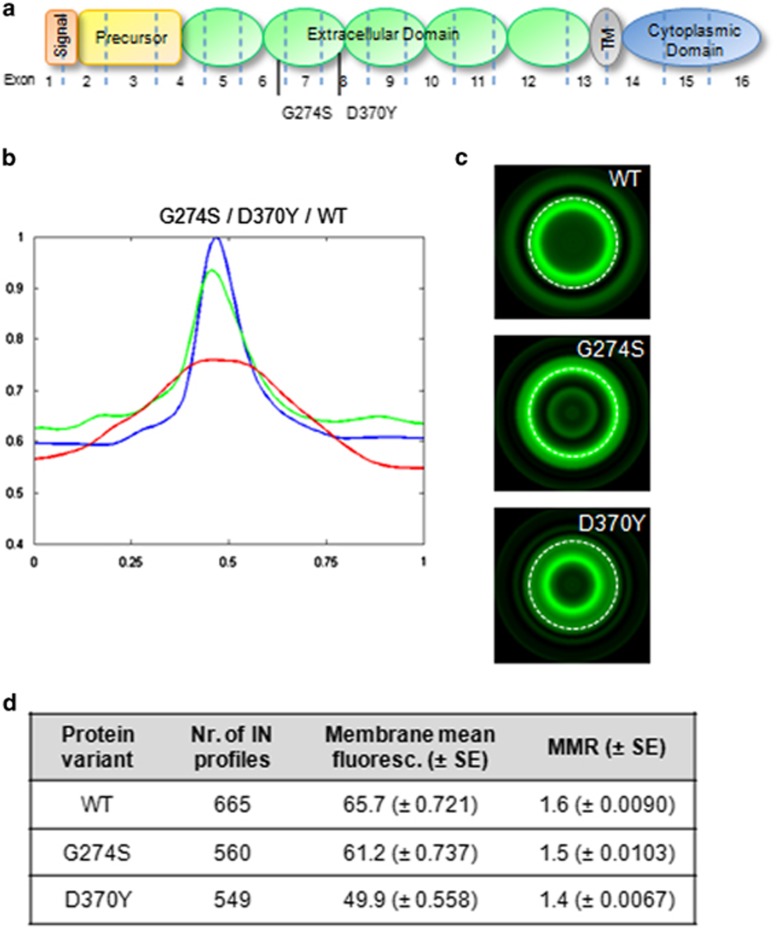
Predictive value of the bioimaging tool. (**a**) Location of the neutral variant c.820G>A (p.Gly274Ser) and the pathogenic c.1108G>T (p.Asp370Tyr). (**b**) IN profiles of WT cells (blue line), cells expressing the neutral variant (green line), and the pathogenic variant (red line). (**c**) Typical virtual cells for WT and E-cadherin variants. (**d**) Quantification of E-cadherin intensity profiles. Mean fluorescence intensity±SE and MMR are presented. All results obtained in cells expressing E-cadherin variants when compared with WT cells are significantly different (Bonferroni-corrected *P*-value<0.017).

To test this hypothesis, we ran a new batch of experiments comprising two different variants that affect the same protein domain—the cadherin repeat 2 (EC2) of the extracellular domain—but display different effects on protein function (Figure 3a). The c.1108G>T (p.Asp370Tyr) variant was considered to be a loss of function variant,^24^ whereas the c.820G>A (p.Gly274Ser) was previously classified as a neutral variant^17^ (Supplementary Table 1 and Table 1).

The protein signatures obtained for both variants were clearly distinct. The neutral variant presents an IN profile superimposed with that of WT cells (Figure 3b and d). The membrane mean fluorescence and MMR values were, respectively, 65.7 and 1.6 for the WT, and 61.2 and 1.5 for the c.820G>A (p.Gly274Ser) variant. Moreover, the corresponding virtual cells also confirmed comparable expression phenotypes: high E-cadherin concentration at the plasma membrane and absence of cytoplasmic protein aggregates (Figure 3c). On the other hand, the c.1108G>T (p.Asp370Tyr) pathogenic variant is scattered across the cell cytoplasm and, consequently, reduced at the plasma membrane (membrane mean fluorescence=49.9, MMR=1.4).

Taken together, these results suggest that this bioimaging tool could be an important complement to assess the pathogenic significance of novel E-cadherin missense variants.

## Discussion

In this study, we describe a bioimaging algorithm that calculates the pattern of expression of a specific protein using *in situ* IF images. This is accomplished by computing a set of quantitative features that can easily discriminate WT from mutated proteins, as perceived for germline E-cadherin variants associated with HDGC.

Currently, a number of methods based on IF images are available for the quantification of cell volumes and analysis of single cell movements.^32^ A class of automatically computed methods was also developed to study a population of cells instead of single cells.^33^ Nevertheless, to study cell populations, it is necessary to circumvent two main difficulties rarely considered: cell heterogeneity and disparity of parameters occurring during image acquisition. In this work, a geometric compensation was performed to deal with cell population heterogeneity. This is of particular importance because E-cadherin alterations may affect cell cytoskeleton organization and consequently cell morphology,^4, 18, 34^ introducing a confounding factor in the analyses. Moreover, cell selection was conducted in a semi-automated form, meaning that intervention of the operator is allowed. Thus, we combine the advantages of the automatism (speed, accuracy, and objectivity) with the expertise of the user. Contrarily to a completely automated system in which the analysis is random and ‘blind', in a semi-automated approach, the user could select the situations with true biological meaning and exclude the ones that might represent technical problems. For example, E-cadherin-negative cells due to protein degradation at the proteasome, or due to technical pitfalls related to transfection efficiency, could be removed from the batch of analysis by the operator.

The analytical pipeline was composed by the following steps: (i) cell selection; (ii) profile extraction and length normalization; (iii) geometrical compensation to cope with cell shape and size variability; (iv) 1D expression profile computation; and (v) 2D virtual cell construction (Figure 1). Data extraction and statistical analysis were then obtained.

The generated IN profiles report in detail the expression level of a protein between two contiguous cells. In addition to quantifying the protein in all points of the sketched line, the IN profiles were also able to translate the pattern of the protein distribution within the cells and classify the sharpness of fluorescence between neighboring cells. In fact, the sharpness of fluorescence at the inter-cellular level, obtained through the quantification of the MMR parameter, is of critical importance in the case of E-cadherin because this feature indirectly measures the tightness of cell–cell adhesion, and thus the function of the protein. Moreover, using this *in situ* evaluation, we guarantee that E-cadherin expression levels and localization are assessed under conditions that allow the exercise of E-cadherin biological functions. Other techniques, namely fluorescence-activated cell sorting were also employed to analyze the fraction of E-cadherin present in the plasma membrane in the context of E-cadherin variants.^12, 14^ Nevertheless, this method is limited to quantification of membrane E-cadherin in a non-adherent situation, as cells need to be in suspension to be analyzed. Under these conditions, the cell–cell adhesion is impaired, and as a consequence E-cadherin levels could be altered.

Herein, using cells expressing WT E-cadherin and a number of variants, we were able to perform an extensive characterization of E-cadherin at the inter-cellular space, at the plasma membrane, and throughout the cytoplasm in all cell lines. More importantly, we were able to discriminate the cells expressing WT or neutral E-cadherin variants from those expressing pathogenic variants. In accordance with our previous results,^12, 14^ we verified that cells expressing E-cadherin pathogenic variants, when compared with WT cells, displayed decreased fluorescence intensity at the membrane, and/or aberrant peaks corresponding to protein accumulation in the perinuclear region (Figure 2, Figure 3, and Table 2). E-cadherin variants, such as c.1018A>G (p.Thr340Ala), could be correctly located at the plasma membrane without aberrant cytoplasmic accumulation of the protein, but still be pathogenic as they present less E-cadherin molecules at the membrane. Besides the impact on cell–cell adhesion and the invasive behavior, this variant also show reduced stability of E-cadherin/EGFR heterodimers and, consequently, increased motile ability (Table 1).^16, 18, 19, 35, 36^

Our group has demonstrated that *CDH1* pathogenic variants are translated into E-cadherin molecules with severe structural abnormalities, leading to protein destabilization and misfolding.^12, 37, 38^ Misfolded proteins are critically regulated by mechanisms of protein quality control, namely endoplasmic reticulum associated degradation, and are degraded by the ubiquitin-proteasome system.^12, 37, 38^ Recently, we have also showed that HDGC variants hamper the binding of key exocytosis-related partners, such as *β*-catenin and PIPKI*γ*, therefore affecting the quantity of E-cadherin molecules trafficked to the membrane.^14, 37^ Variants affecting the p120-binding domain (p.Arg749Trp, p.Glu757Lys, and p.Glu781Asp) block the E-cadherin/p120-catenin interplay and, as a consequence, these mutant proteins become more available to be targeted by Hakai for ubiquitination and to be degraded.^14^ Interestingly, all these posttranslational regulation mechanisms culminate with premature degradation of E-cadherin, and thus it is now well established that low total and surface E-cadherin expression is frequently observed in the presence of E-cadherin missense variants when compared with the WT cells.^12, 14, 37, 38^ Despite the differences at protein level, *CDH1* mRNA is similar in WT cells and in cells expressing the *CDH1* variants, demonstrating that protein loss is not a transfection artifact.^12^

To further assist the recognition of an abnormal pattern of E-cadherin expression, we studied the fluorescence intensity in single selected cells by designing a large number of RD profiles with center at the nuclei of a cell (data not shown). This strategy enables not only the quantification and mapping of E-cadherin within a single cell, but also the construction of a virtual cell representing the complete E-cadherin signature (Figure 2b). Using this approach, we verify that each E-cadherin variant exhibits a particular pattern of E-cadherin spatial distribution that can represent different stages of trafficking dynamics and, consequently, accumulation of the mutant proteins in distinct cell compartments. Indeed, we have previously reported that each missense variant behaves in a singular way, interacting differently with its binding partners and playing different roles in signal transduction.^14^

Herein, we demonstrate that our bioimaging approach is a powerful tool to assist in the identification of functionally relevant missense variants, and thus, it should be used in combination with the classical *in vitro* functional assays^13, 14, 38^ for genetic screening. We propose that our methodology can be used in a computer aid diagnosis framework for semi-automatic detection/screening of dysfunctional proteins to diagnostic and therapeutic evaluation purposes, not only in cancer but also in other diseases involving abnormal expression or localization of a specific protein.

## Figures and Tables

**Table 1:** *In vitro* features of E-cadherin missense variants

*Protein variant*	*Genetic alteration*	*Domain*	*Cell-cell adhesion*	*Invasion*	*Motility*	*Catenin complex assembly*	*Surface E-cadherin*	*Total E-cadherin*	*Trafficking defects*	*EGFR activation*	*References*
Wild type	—	—	Compact aggregates	No	No	Yes	Regular	Regular	No	No	
p.Gly274Ser	c.820G>A	EC2	Compact aggregates	No	Not studied	Not studied	Regular	Regular	Not studied	Not studied	^17^
p.Thr340Ala	c.1018A>G	EC2	Small aggregates	Yes	Yes	Yes	Regular	Regular	Not found yet	Yes	^13, 14, 18, 19, 35, 36, 40^
p.Asp370Tyr	c.1108G>T	EC2	isolated phenotype	Yes	Not studied	Not studied	Reduced	Regular	Not studied	Not studied	^24^
p.Ala634Val	c.1901C>T	EC5	Small aggregates	Yes	Yes	Yes	Reduced	Reduced	**↓** exocytosis	Yes	^13, 14, 18, 35, 36, 40^
p.Arg749Trp	c.2245C>T	Juxtamemb	Small aggregates	Yes	Yes	No	Reduced	Reduced	↓ exocytosis**↑** endocytosis	Yes	^12, 14, 16, 35, 37^
p.Glu757Lys	c.2269G>A	Juxtamemb	isolated phenotype	Yes	Yes	No	Reduced	Reduced	↓ exocytosis↑ endocytosis	Yes	^12, 14, 35, 37^
p.Glu781Asp	c.2343A>T	Intracellular	isolated phenotype	Yes	No	No	Reduced	Reduced	↓ exocytosis↑ endocytosis	No	^14, 16, 36, 41^
p.Pro799Arg	c.2396C>G	Intracellular	Small aggregates	Yes	No	Not studied	Reduced	Reduced	↓ exocytosis	No	^14, 21, 35, 36, 40^
p.Val832Met	c.2494G>A	Intracellular	Small aggregates	Yes	No	No	Reduced	Regular	↓ exocytosis	No	^14, 18, 35, 36, 39, 40^

For each E-cadherin variant, the corresponding nucleotide change and affected domain is presented. *In vitro* behavior concerning cell–cell aggregation, invasive ability, and motility is described. E-cadherin profile regarding surface and total expression, adhesion complex assembly, as well as trafficking defects and EGFR activation is also displayed.

**Table 2:** Quantification of E-cadherin profiles

*Protein variant*	*Number of IN profiles*	*Membrane mean fluorescence (±SE)*	*MMR (±SE)*
WT	670	105.8 (±0.834)	1.6 (±0.0076)
p.Thr340Ala	600	75.7 (±0.640)	1.4 (±0.0055)
p.Ala634Val	980	67.3 (±0.631)	1.5 (±0.0071)
p.Arg749Trp	780	79.5 (±1.435)	1.5 (±0.0089)
p.Glu757Lys	920	68.5 (±0.550)	1.3 (±0.0046)
p.Glu781Asp	918	57.8 (±0.835)	1.4 (±0.0056)
p.Pro799Arg	720	66.8 (±0.725)	1.3 (±0.0054)
p.Val832Met	576	71.9 (±1.129)	1.4 (±0.0062)

Mean and standard error (SE) of the fluorescence intensity for WT and E-cadherin variants. MMR quantifies the sharpness of the fluorescence peak at the membrane. All results obtained in cells expressing E-cadherin variants when compared with WT cells are significantly different (Bonferroni-corrected *P*-value<0.002).
