# Supplementary material for: Quantification of mutant E-cadherin using bioimaging analysis of in situ fluorescence microscopy. A new approach to CDH1 missense variants
Source: Eur J Hum Genet. 2014 Nov 12;23(8):1072–9. doi: 10.1038/ejhg.2014.240 (PMC4795115; doi:10.1038/ejhg.2014.240)
Supplement: Supplementary Table 1 [file ejhg2014240x1.doc]

**SUPPLEMENTAL MATERIAL**

**Supplemental Table 1. Clinical and *in silico* data of E-cadherin missense variants.** For each E-cadherin variant, clinical data such as disease presentation, segregation studies, recurrence in independent families, and frequency in control population is shown. Aminoacid conservation, SIFT prediction, *in vitro* functional classification, as well as the combined evaluation of missense variants are provided. (GC) sporadic gastric cancer. (CLP) cleft lip with or without cleft palate. (ESP) Exome Sequencing Project database, generated from approximately 2500 exomes.

| **Protein variant** | **Clinical presentation** | **Segregation** | **Recurrence** | **Frequency in control population** | **AA conservation** | ***SIFT*** | ***In vitro* classification** | **Combined classification** | **Refer.** |
| --- | --- | --- | --- | --- | --- | --- | --- | --- | --- |
| **p.Gly274Ser** | Sporadic GC | - | - | 0/108 | High | Intolerant | Neutral | Neutral | [^17^](#_ENREF_17) |
| **p.Thr340Ala** | HDGC | No | Yes | 1/50 | Low | Tolerant | Deleterious | Deleterious |  |
| **p.Asp370Tyr** | CLP | No | - | Not in ESP | High | Intolerant | Deleterious | Deleterious | [^24^](#_ENREF_24) |
| **p.Ala634Val** | HDGC | No | Yes | 1/100 | Low | Tolerant | Deleterious | Deleterious |  |
| **p.Arg749Trp** | HDGC | - | No | Not determined | High | Intolerant | Deleterious | Deleterious | [^16^](#_ENREF_16) |
| **p.Glu757Lys** | HDGC | Yes | No | 0/100 | High | Intolerant | Deleterious | Deleterious | [^12^](#_ENREF_12) |
| **p.Glu781Asp** | HDGC | - | Yes | Not determined | Low | Tolerant | Deleterious | Deleterious |  |
| **p.Pro799Arg** | HDGC | No | No | 0/50 | High | Intolerant | Deleterious | Deleterious |  |
| **p.Val832Met** | HDGC | Yes | No | Not determined | High | Intolerant | Deleterious | Deleterious |  |
